# From Lesions to Networks: Defining the Causal Circuitry of Obsessive-Compulsive Disorder

**DOI:** 10.1192/j.eurpsy.2026.10643

**Published:** 2026-07-31

**Authors:** G. Cotovio, N. Descalço, J. Caballero-Insaurriaga, D. R. Martins, F. Faro Viana, C. Fonseca, J. Ramos, B. Santos, A. Maia, J. Oliveira, N. Loução, T. Barbour, S. H. Siddiqi, M. D. Fox, J. B. Barahona-Corrêa, A. J. Oliveira-Maia

**Affiliations:** 1 Champalimaud Foundation, Lisbon, Portugal; 2 Harvard Medical School, Boston, United States

## Abstract

**Introduction:**

We aimed to define the topography of brain lesions leading to OCD, identify functional brain networks specifically connected to such lesions, and examine convergence of the lesional OCD network with idiopathic OCD pathophysiology and with treatment targets for transcranial magnetic stimulation (TMS) and deep brain stimulation (DBS).

**Objectives:**

We aimed to (i) define the topography of brain lesions leading to OCD, (ii) identify functional brain networks specifically connected to such lesions, and (iii) examine convergence of the lesional OCD network with idiopathic OCD pathophysiology and with treatment targets for transcranial magnetic stimulation (TMS) and deep brain stimulation (DBS).

**Methods:**

We systematically reviewed the literature and identified 123 cases of lesional OCD. Forty cases had traceable lesion images, which were mapped onto a standard brain template. Topography was analyzed against a large control lesion cohort (N=608; tumors and strokes). Lesion network mapping was performed using normative resting-state fMRI connectomes (N=1000). Reliability was tested with inter-/intra-rater lesion tracings and alternative connectomes. Robustness was assessed in multiple sensitivity analyses. Convergence with idiopathic OCD was evaluated by comparing the lesional OCD network with Neurosynth-derived OCD maps, approved TMS/DBS targets, a TMS improvement network (N=14), and resting-state fMRI from a case-control study (N=54 OCD, 61 healthy controls).

**Results:**

Lesional OCD topography localized predominantly to the orbitofrontal cortex and temporal poles compared to control lesions. Network mapping revealed consistent connectivity from lesional sites to bilateral orbitofrontal cortex and basal ganglia. These findings were robust across reliability and sensitivity analyses. Lesional OCD connectivity showed selective overlap with Neurosynth OCD maps, but not with other syndromes. The network further converged with established TMS and DBS targets, and overlapped significantly with a TMS improvement network. In idiopathic OCD, individual functional connectivity to the lesional OCD network differed between patients and healthy controls, particularly in frontal and striatal regions. Exploratory analysis suggests treatment-related connectivity normalization in responders.

**Conclusions:**

Lesional OCD lesions localize to orbitofrontal-temporal cortices but converge on a broader causal network involving orbitofrontal and basal ganglia circuits. This network aligns with functional alterations in idiopathic OCD and overlaps with clinically effective neuromodulation targets. Our findings provide causal evidence for the brain circuits underlying OCD and support network-guided optimization of TMS and DBS interventions.

**Disclosure of Interest:**

G. Cotovio Grant / Research support from: NARSAD 2023 Young Investigator Grant; Proof-of-Concept Grant from the European Research Council, N. Descalço Grant / Research support from: FCT through PhD Scholarships, J. Caballero-Insaurriaga: None Declared, D. Martins: None Declared, F. Faro Viana: None Declared, C. Fonseca: None Declared, J. Ramos: None Declared, B. Santos: None Declared, A. Maia Grant / Research support from: FCT through PhD Scholarships, J. Oliveira Grant / Research support from: NARSAD 2018 Young Investigator Grant, N. Loução Employee of: Philips Healthcare, Portugal., T. Barbour: None Declared, S. Siddiqi Grant / Research support from: Sidney R. Baer and Brain & Behavior Research Foundations, M. Fox Grant / Research support from: Sidney R. Baer Jr. Foundation, the NIH (R01MH113929 R01MH113929, R21MH126271, R56AG069086, R21NS123813), the Nancy Lurie Marks Foundation, the Kaye Family Research Fund, the Ellison/Baszucki Foundation, and the Mather’s Foundation, J. B. Barahona-Corrêa Grant / Research support from: FCT-PTDC/MEC-PSQ/30302/2017-IC&DT-LISBOA-01-0145-FEDER, funded by national funds from FCT/MCTES and co-funded by FEDER, under the Partnership Agreement Lisboa 2020 - Programa Operacional Regional de Lisboa, Consultant of: JBB-C received honoraria in 2018 as member of the local Advisory Board for Trevicta from Janssen-Cilag, Ltd., A. Oliveira-Maia Grant / Research support from: Proof-of-Concept Grant from the European Research Council; FCT-PTDC/MEC-PSQ/30302/2017-IC&DT-LISBOA-01-0145-FEDER, funded by national funds from FCT/MCTES and co-funded by FEDER, under the Partnership Agreement Lisboa 2020 - Programa Operacional Regional de Lisboa; Schuhfried GmBH for norming and validation of cognitive tests; National coordinator for Portugal of trials of psilocybin therapy for treatment-resistant depression, sponsored by Compass Pathways, Ltd (EudraCT number 2017-003288-36 and 2020-001348-25), and of esketamine for treatment-resistant depression, sponsored by Janssen-Cilag, Ltd (EudraCT NUMBER: 2019-002992-33;, Consultant of: AJOM has received payment, honoraria, or support for attending meetings and participating in advisory boards from MSD, Neurolite AG, Janssen Pharmaceuticals, Angelini Pharma, and the European Monitoring Centre for Drugs and Drug Addiction; has received consultancy fees from Bioprojet Pharma and NaturalX Health Ventures (all outside the submitted work); is Vice President of the Portuguese Society for Psychiatry and Mental Health; is head of the Psychiatry Working Group for the National Board of Medical Examination at the Portuguese Medical Association and Portuguese Ministry of Health; is President of the Ethics Committee for the Public Institute for Addictive Behaviors and Dependence; and is President of the Scientific Council of the Portuguese Obsessive Compulsive Disorder Foundation.

